# EGFR-TKIs与化疗比较一线治疗非小细胞肺癌疗效的*meta*分析

**DOI:** 10.3779/j.issn.1009-3419.2015.03.04

**Published:** 2015-03-20

**Authors:** 飞飞 曹, 琳琳 张, 双 王, 殿胜 钟, 燕 王

**Affiliations:** 1 300052 天津，天津医科大学总医院呼吸内科 Department of Respiratory Medicine, Tianjin Medical University General Hospital, Tianjin 300052, China; 2 300052 天津，天津医科大学总医院肿瘤科 Department of Medical Oncology, Tianjin Medical University General Hospital, Tianjin 300052, China

**Keywords:** EGFR-TKI, 肺肿瘤, 化疗, 一线治疗, *Meta*分析, EGFR-TKIs, Lung neoplasms, Chemotherapy, First-line treatment, *Meta*-analysis

## Abstract

**背景与目的:**

表皮因子受体酪氨酸激酶抑制剂（epidermal growth factor receptor tyrosine kinase inhibitor, EGFR-TKI）应用于非小细胞肺癌（non-small cell lung cancer, NSCLC）一线治疗取得较好的临床疗效，然而EGFR-TKI一线用药选择仍面临很多问题。本研究应用循证医学的方法对NSCLC患者临床特征及基因突变情况对一线EGFR-TKIs治疗及化疗获益进行分析，以便指导临床用药。

**方法:**

用计算机检索Pubmed、Embase、American Society of Clinical Oncology（ASCO）、European Society for Medical Oncology（ESCO）及生物医学数据库等数据库，寻找出EGFR-TKI与化疗相比一线治疗NSCLC的疗效的随机对照研究（randomized controlled trials, RCT）。对纳入RCT进行资料提取和质量评价，采用Review Manager 5.2软件分析、对比NSCLC患者在TKI治疗中的疗效。

**结果:**

纳入的14项研究，共5, 000例患者。*Meta*分析结果显示，*EGFR*基因突变的NSCLC患者，EGFR-TKIs治疗与化疗相比有较好的近期有效率（RR=2.31; 95%CI: 1.88-2.84）和延长无疾病进展时间（progression free survival, PFS）（HR=0.39; 95%CI: 0.30-0.49），总生存时间（overall survival, OS）上两者无明显差异（HR=0.99; 95%CI: 0.84-1.16）。临床选择（亚裔、腺癌、不吸烟）NSCLC患者，EGFR-TKIs一线治疗与化疗相比也有较好的近期有效率（RR=1.30; 95%CI: 1.15-1.47），PFS和OS无差异（HR=0.93; 95%CI: 0.58-1.49）（HR=0.91; 95%CI: 0.81-1.02）。未经选择的患者，一线EGFR-TKIs治疗有效率、PFS与化疗无差异，但OS劣于一线接受化疗患者。

**结论:**

*EGFR*突变的NSCLC患者一线EGFR-TKIs获益更多；对于不能耐受化疗的亚裔、腺癌、不吸烟患者，推荐一线EGFR-TKIs治疗；未经选择的NSCLC患者一线EGFR-TKIs治疗临床无获益，而且一线EGFR-TKI治疗的OS明显低于一线化疗。

## Abstract (en)

**Background and objective:**

Epidermal growth factor receptor tyrosine kinase inhibitor (EGFR-TKI) has been used for the first-line treatment of non-small cell lung cancer (NSCLC) and has shown good clinical effects. However, some patients fail to benefit from this treatment. The aim of this study is to analyze whether or not clinical-selected patients (Asian, adenocarcinoma histology, non-smoking) and *EGFR* mutation-selected patients benefit from EGFR-TKIs or chemotherapy. Our results could be used as basis to guide clinical therapy.

**Methods:**

Randomized controlled trials evaluating the efficacy of EGFR-TKIs versus chemotherapy as first-line treatments of NSCLC were obtained from electronic databases, namely, PubMed, Embase, American Society of Clinical Oncology (ASCO), European Society for Medical Oncology, and China Biology Medicine disc. Assessment, data collection, and statistical analysis were performed according to Cochrane Handbook 5.1.0.

**Results:**

A total of 14 randomized controlled trials with 5, 000 patients were included in this study. Compared with the chemotherapy group, EGFR-TKI therapy group in *EGFR* mutation-selected NSCLC patients showed a higher response rate (RR=2.31; 95%CI: 1.88-2.84) and more significant improvement in progression free survival (PFS; HR=0.39; 95%CI: 0.30-0.49); by contrast, no significant difference was observed in overall survival (OS; HR=0.99; 95%CI: 0.84-1.16). The response rate of clinical-selected patients treated with EGFR-TKI significantly increased (RR=1.30; 95%CI: 1.15-1.47) compared with that of the patients treated with chemotherapy; PFS (HR=0.93; 95%CI: 0.58-1.49) and OS (HR=0.91; 95%CI: 0.81-1.02) of the two groups did not significantly differ. Likewise, the PFS and the OS of the unselected patients in the EGFR-TKI treatment group and the chemotherapy group did not significantly differ, although the OS of the former was shorter than that of the latter.

**Conclusion:**

*EGFR* mutation-selected patients received more benefits from EGFR-TKI first-line treatment than other treatments. First-line EGFR-TKI treatment was recommended for clinically selected patients who were unsuitable for themotherapy. By comparison, first-line EGFR-TKI treatment was not a suitable choice for unselected patients.

肺癌的死亡率仍居于各类癌症之首^[1]^，其中约85%为非小细胞肺癌（non-small cell lung cancer, NSCLC），约65%的患者就诊时已属晚期^[2]^。目前，含铂双药化疗方案仍为一线治疗晚期NSCLC的标准方案，但NSCLC的中位生存期仅为8个月-10个月^[3]^。表皮生长因子酪氨酸激酶抑制剂（epidermal growth factor receptor tyrosine kinase inhibitor, EGFR-TKI）作为一种治疗NSCLC的分子靶向药物，具有高效低毒的优势，为NSCLC的治疗指出了一个新的方向。

吉非替尼是首个被应用于临床的EGFR-TKI，EGFR-TKI是一种小分子药物，它通过与ATP竞争结合于细胞膜表皮生长因子受体的酪氨酸区域，阻止酪氨酸磷酸化，抑制细胞内一系列与肿瘤细胞的形成、增殖、凋亡相关的信号通路联级反应，从而抑制肿瘤细胞的增殖^[4, 5]^。早在2002年进行的Ⅰ期临床研究就让研究者们了解到实体瘤及NSCLC患者能耐受该药，且部分NSCLC患者能从中获益^[6, 7]^，接着通过大型的Ⅱ期临床研究（IDEAL），研究者们看到了该药11.8%到18.4%的近期有效率和较小的副作用^[8, 9]^。随后对厄洛替尼和阿法替尼的研究得出跟吉非替尼相似的抗肿瘤效果^[10]^。

IDEAL研究中，研究者已经发现亚裔、腺癌、不吸烟的患者对EGFR-TKIs有更好的近期有效率^[8, 9]^。随后Lynch和他的团队^[11]^对EGFR的整个编码区域进行了测序，让人们更精准的了解拥有EGFR酪氨酸激酶区域杂合性错义突变的患者对EGFR-TKIs有更好的近期有效率，之后大量的临床研究也证实了*EGFR*基因突变的NSCLC患者在EGFR-TKIs治疗中获益颇多，尽管最终NSCLC美国国立综合癌症网络（National Comprehensive Cancer Network, NCCN）指南提出了在*EGFR*突变的患者中应用EGFR-TKIs^[12]^，但EGFR-TKIs在临床应用上仍面临很多问题，譬如*EGFR*突变的患者一线EGFR-TKIs治疗是否比含铂两药方案化疗获益更多；*EGFR*基因无突变的NSCLC患者若不能耐受化疗，能否从EGFR-TKIs治疗中获益，这部分患者能否通过患者临床特征（亚裔、腺癌、不吸烟）进行筛选用药；不能进行*EGFR*基因突变分析的患者一线能否从EGFR-TKIs治疗中获益等。为了使EGFR-TKIs在临床应用中发挥出最大的优势，本研究用*meta*分析的方法，对近年来EGFR-TKIs与化疗比较一线治疗临床选择的（亚裔、腺癌、不吸烟）、非选择的及*EGFR*基因突变的NSCLC患者的疗效的临床研究进行综合定量分析，以更好地指导EGFR-TKIs在临床中的应用。

## 1 资料和方法

### 1.1 研究资料

#### 1.1.1 研究类型

随机对照（randomized controlled trials, RCT）Ⅱ期或Ⅲ期临床试验，不论是否使用盲法。

#### 1.1.2 研究对象

有组织学或细胞学证实的局部晚期或可控的转移性NSCLC患者（包括*EGFR*基因突变患者，临床选择患者即亚裔、腺癌、不吸烟患者，未选择患者）；年龄＞18岁；既往未接受过系统的抗肿瘤治疗；无严重的血液学、生物化学及器官功能障碍。

#### 1.1.3 干预措施

实验组使用任意一种EGFR-TKIs（吉非替尼、厄洛替尼、阿法替尼、埃克替尼），对照组使用化疗。

#### 1.1.4 结局指标

主要结局指标为无疾病进展时间（progression free survival, PFS），即患者从接受治疗开始到观察到疾病进展或者发生因为任何原因的死亡之间的这段时间；有效率，即完全缓解（complete response, CR）与部分缓解（partial response, PR）之和除以总例数。次要结局指标为总生存时间（overall survival, OS），即从随机化分组开始至因任何原因引起死亡的时间。

### 1.2 方法

#### 1.2.1 检索策略

以“gefitinib”、“erlotinib”、“icotinib”、“afatinib”、“non small lung cancer”、“chemotherapy”、“EGFR mutation”、“never smokers”、“adenocarcinoma”、“clinical selected”为关键词，在Pubmed、Embase、American Society of Clinical Oncology（ASCO）、European Society for Medical Oncology（ESCO）及生物医学数据库中检索近10年来EGFR-TKI与化疗相比一线治疗NSCLC疗效的相关文献。

#### 1.2.2 资料提取

对纳入研究的基本信息进行提取，包括EGFR-TKIs具体药物及剂量、化疗具体方案、实验组及对照组人数、各组患者特点、随访时间、该研究所在地区。对纳入研究的结局数据进行提取，包括肿瘤反应率、PFS和OS的风险比（hazard ratio, HR）及95%CI。

#### 1.2.3 质量评价

纳入研究的方法学质量评价采用Cochrane Reviewer Handbook 5.2.0中RCT的偏倚风险评价标准，包括：随机是否充分、隐蔽分组是否充分、对参与者和实施者是否使用盲法、对结局评价是否使用盲法、结局数据是否完整、是否有选择性发表偏倚、是否有其他偏倚。针对上述标准，采用低偏倚风险、高偏倚风险和缺乏相关信息或偏倚情况不确定进行分级。

### 1.3 统计学方法

采用Review Manager 5.2软件进行统计学分析和森林图的制作。肿瘤反应率的数据类型为二分类变量（Dichotomous），以相对危险度RR（relative risk）为效应指标。PFS与OS的资料统计分析方法为倒方差法（generic inverse variance），以HR为效应指标。各效应指标均以95%CI表示。在效应指标中*P*＜0.05或95%CI上下限不包含1表示有统计学意义，HR＞1表示接受EGFR-TKIs治疗与化疗比较有更多的患者死亡或疾病进展，RR＞1表示接受EGFR-TKIs治疗与化疗比较有更好的有效率。表示各研究的异质性采用*I*^2^检验，若*I*^2^≤50%，表明各研究间统计学异质性较小，采用固定效应模型进行分析；若*I*^2^＞50%，则表明各研究间存在较大异质性，先分析异质性来源，可采用随机效应模型合并效应量进行分析。

## 2 结果

### 2.1 文献检索结果

通过上述检索策略进行检索后，共检索到相关文献152篇，经阅读题目及摘要，剔除与主题无关及重复文献90篇，再排除二线及维持治疗研究、Ⅰ期临床研究、EGFR-TKIs与安慰剂对照研究、EGFR-TKIs与化疗联合治疗研究46篇。最终纳入16篇文献。该16篇文献中，重复研究的文献有2篇，即16篇文献为14项临床研究，因为该2篇重复文献^[20, 22]^后续结局报道，所以亦纳入研究。

### 2.2 纳入研究基本特征

纳入的14项研究（16篇文献）^[13-28]^，共包含EGFR-TKIs组患者2, 339例，化疗组患者2, 041例。其中5项研究^[13-17]^并未对患者人群进行选择；2项研究为针对临床选择人群的研究，在这2项研究中又分出*EGFR*基因突变阳性亚组进行研究^[18-20]^；其余7项研究为针对*EGFR*基因突变阳性人群的研究^[21-28]^。6项研究的实验组治疗药物为吉非替尼^[14, 17-23]^，5项研究实验组治疗药物为厄洛替尼^[13, 15, 16, 24, 28]^，3项研究实验组治疗药物为阿法替尼^[25-27]^。化疗药物有顺铂+吉西他滨、卡铂+吉西他滨、卡铂+紫杉醇、顺铂+多西他赛、顺铂+培美曲塞、吉西他滨单药、多西他赛单药、长春瑞滨单药。其中一个研究为3组对照研究，该研究被分解为两部分进行分析。纳入分析个研究基本特征见表 1。

**1:** 纳入研究基本特征 Characteristics of included trials

Study	EGFR-TKIs Chemo	Subgroup	Adenocarc inoma (%)	*n*	Median age (years)	Disease stage	EGOG PS	Region	Never smoked (%)	Female (%)	Follow-ing up time
Chen YM^[13]^	Erlotinib Vinorelbine	Unselected	63.2 66.1	57 56	78.1 77.8 (mean)	Ⅲb/Ⅳ	0-3	Taiwan	21 21.4	17.5 19.6	36 mo
Crino L (INVITE)^[14]^	Gefitinib Vinorelbine	Unselected	35.1 45.5	97 99	74 74	Ⅲb/Ⅳ	0-2	Eropean	17.5 11.1	22.7 26.3	19 mo
Gridelli C (TORCH)^[15]^	Erlotinib cisplatin plus gemcitabine	Unselected	55.8 55.3	380 380	63 62	Ⅲb/Ⅳ	0-1	Italy, Canada	20.8 20.5	33.7 33.7	54 mo
LilenbaumR^[16]^	Erlotinib carboplatin plus paclitaxel	Unselected	17 16	52 51	> 70 (46%)	Ⅲb/Ⅳ	2	America	12 8	56 45	NA
Morere JF IFCT-0301)^[17]^	Gefitinib gecitabine docetaxel	Unselected	51 50 45	43 42 42	70 71 71	Ⅲb/Ⅳ	2-3	France	4.7 0 14.3	12 19 21	NA
Han JY (First-SIGNAL)^[18]^	Gefitinib cisplatin plus gemcitabine	Clinical and mutation selected	100 100	159, 26 150, 16	57 56.5	Ⅲb/Ⅳ	0-2	Korea	100 100	88 89.3	49.4 mo
Mok TS (IPASS)^[19, 20]^	Gefitinib carboplatin plus paclitaxel	Clinical and mutation selected	95.4 97.2	609, 132 608, 129	57 57	Ⅲb/Ⅳ	0-2	Asia	100 100	79.5 79.1	51 mo
Maemondo M (NEJ002)^[21, 22]^	Gefitinib carboplatin plus paclitaxel	Mutation selected	90.4 96.5	114 114	63.9 62.6 (mean)	Ⅲb/Ⅳ	0-2	North-east Japan	65.8 57.9	63.2 64	42 mo
Mitsudomi (WJFOG3405)^[23]^	Gefitinib cisplatin puls docetaxel	Mutation selected	96, 5 97.6	86 86	64 64	Ⅲb/Ⅳ	0-1	West Japan	70.9 66.2	68.6 69.7	41 mo
Rosell R (EURTAC)^[24]^	Erlotinib cisplatin puls docetaxel	Mutation selected	95 90	86 87	63.44 64.15	Ⅲ/Ⅳ	0-2	Spanish	66 72	67 78	29 mo
Schuler M^[25]^	Afatinib cisplatin plus pemetrexed	Mutation selected	NA	184 89	61 62	NA	0-1	NA	NA	74 66	NA
Wu YL (LUX-Lung 6^[26]^	Afatinib cisplatin plus gemcitabine	Mutation selected	NA	242 122	58 58	Ⅲb/Ⅳ	0-1	Asian	74.8 81.1	64 68	23 mo
Yang J CH (LUX-Lung 3)^[27]^	Afatinib cisplatin plus pemetrexed	Mutation selected	NA	230 115	61 61	Ⅲb/Ⅳ	0-1	NA	NA	NA	NA
Zhou CC (OPTIMAL) ^[28]^	Erlotinib carboplatin plus gecitabine	Mutation selected	88 86	82 72	57 59	Ⅲb/Ⅳ	0-2	China	72 69	59 60	29 mo
ECOG PS: Eastern Cooperative Oncology Group performance status; NA: not available; EGFR-TKI: epidermal growth factor receptor tyrosine kinase inhibitor.											

### 2.3 纳入研究的质量评价

依据Cochrane Reviewer Handbook 5.2.0中RCT的偏倚风险评价标准，纳入的14项研究中，5项研究^[15, 23, 24, 26, 28]^充分随机及分配隐藏，其余9项研究^[13, 14, 16-22, 25, 27]^随机及分配隐藏方法描述不清楚。对参与者及实施者的盲法方面，并没有研究使用盲法，但鉴于临床试验本身的特点，并不会对研究造成过大偏倚；在对结局评价实施盲法方面，12项研究并未描述此情况，1项研究未实施盲法^[28]^，1项研究实施了盲法^[26]^。6项研究^[13, 16, 17, 25, 27, 28]^并未报道结局指标总生存时间。1项研究^[26]^由于患者基线水平体能状态（performance status, PS）评分在两组随机分配时并不平衡，有可能导致偏倚（图 1）。

**1:**
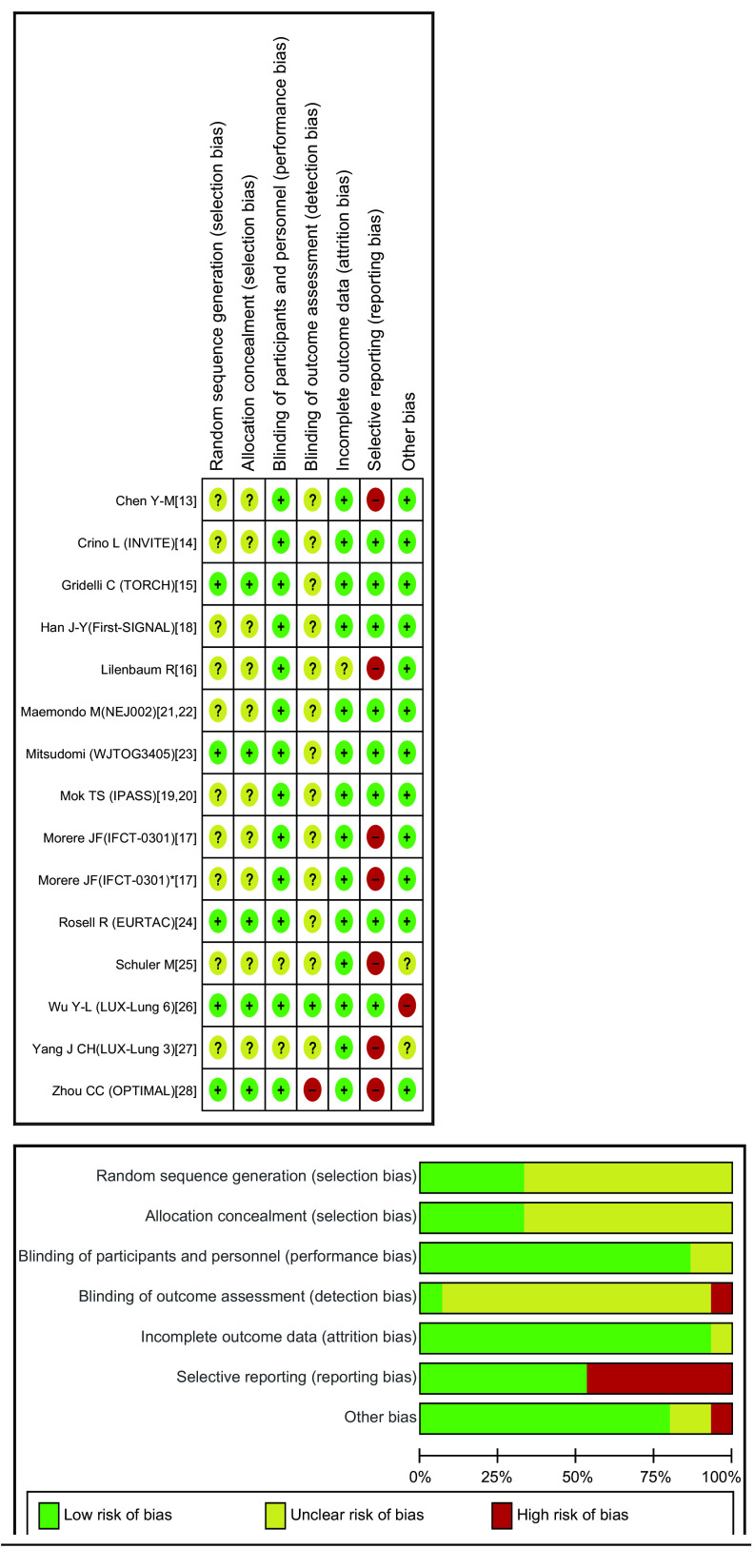
纳入研究的偏倚风险图 Risk of bias of included trials. -: High risk of bias; ?: Unclear risk of bias; +: Low risk of bias.

### 2.4 *Meta*分析结果

#### 2.4.1 有效率的*meta*分析结果

EGFR-TKIs治疗组的有效率为0-84.6%，化疗组有效率为5.1%-47.3%。*Meta*分析结果显示，总研究存在异质性（Chi^2^=76.98, *P*＜0.000, 01, *I*^2^=79%），采用随机效应模型。EGFR-TKIs治疗组与化疗组比有更好的肿瘤有效率（RR=1.97; 95%CI: 1.59-2.44）。在亚组分析中，临床选择组与*EGFR*基因突变阳性选择组EGFR-TKIs治疗比化疗有更好的有效率（RR=1.30; 95%CI: 1.15-1.47）、（RR=2.31; 95%CI: 1.88-2.84）。而对于未选择人群，EGFR-TKIs治疗与化疗相比有效率并未显示出优势（RR=1.04; 95%CI: 0.41-2.62）（图 2）。

**2:**
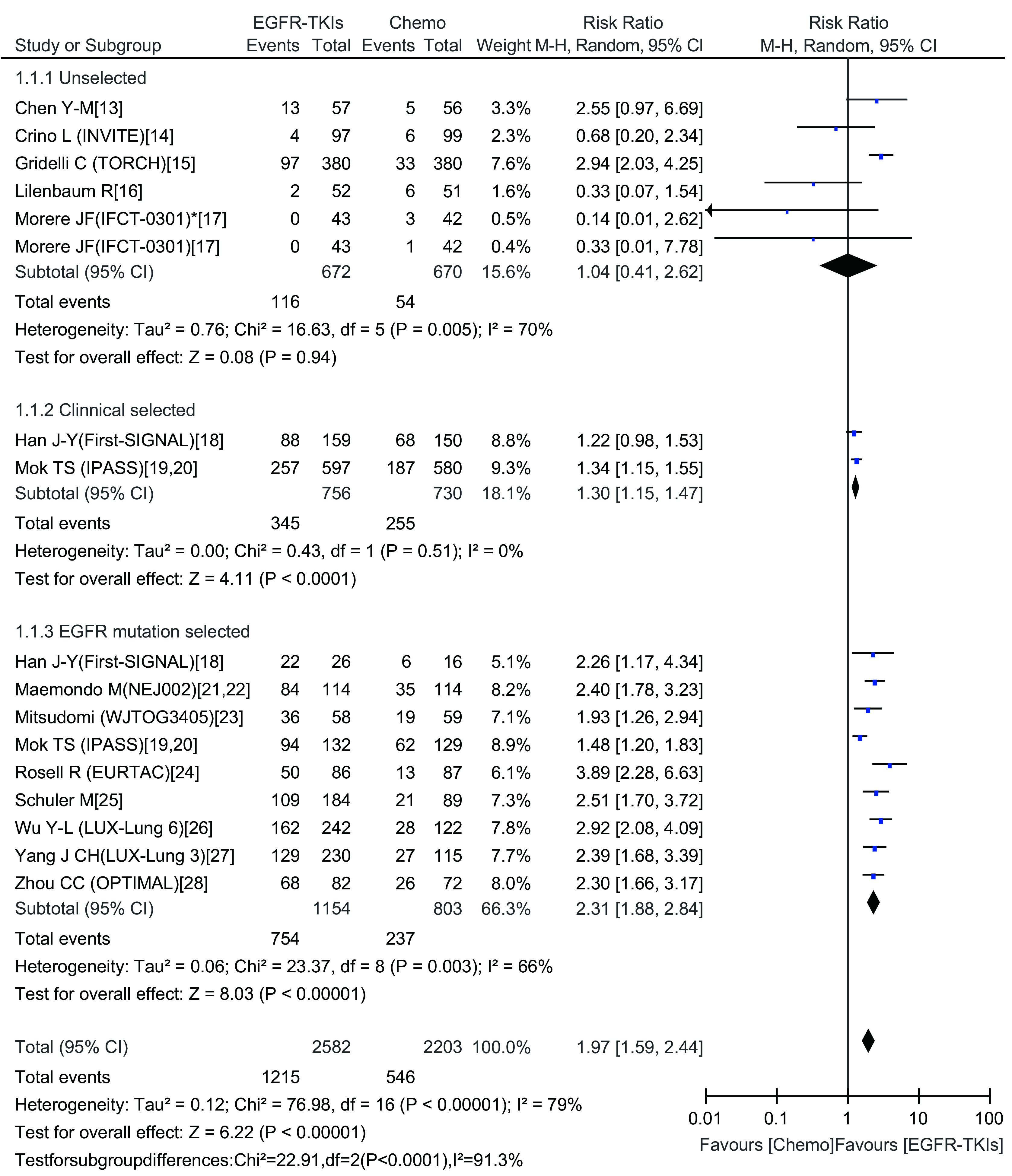
一线EGFR-TKIs治疗与化疗比较有效率的森林图及统计学分析 Forest plot and statistics of first-line EGFR-TKI treatment versus chemotherapy for response rate. EGFR-TKI: epidermal growth factor receptor tyrosine kinase inhibitor.

#### 2.4.2 PFS的*meta*分析结果

EGFR-TKIs治疗组与化疗组的中位PFS分别为1.9个月-13.7个月、2.0个月-8.1个月。*Meta*分析结果显示，总研究及各亚组均存在异质性（Chi^2^=211.90, *P*＜0.000, 01, *I*^2^=92%），采用随机效应模型。EGFR-TKIs治疗与化疗比延长了PFS（HR=0.59; 95%CI: 0.46-0.77）。但在各亚组分析中，只有E*GFR*基因突变阳性患者接受EGFR-TKIs治疗比化疗更有优势（HR=0.39; 95%CI: 0.30-0.49），其余两组两种治疗方法之间无差异（HR=0.97; 95%CI: 0.75-1.25）、（HR=0.93; 95%CI: 0.58-1.49）（图 3）。

**3:**
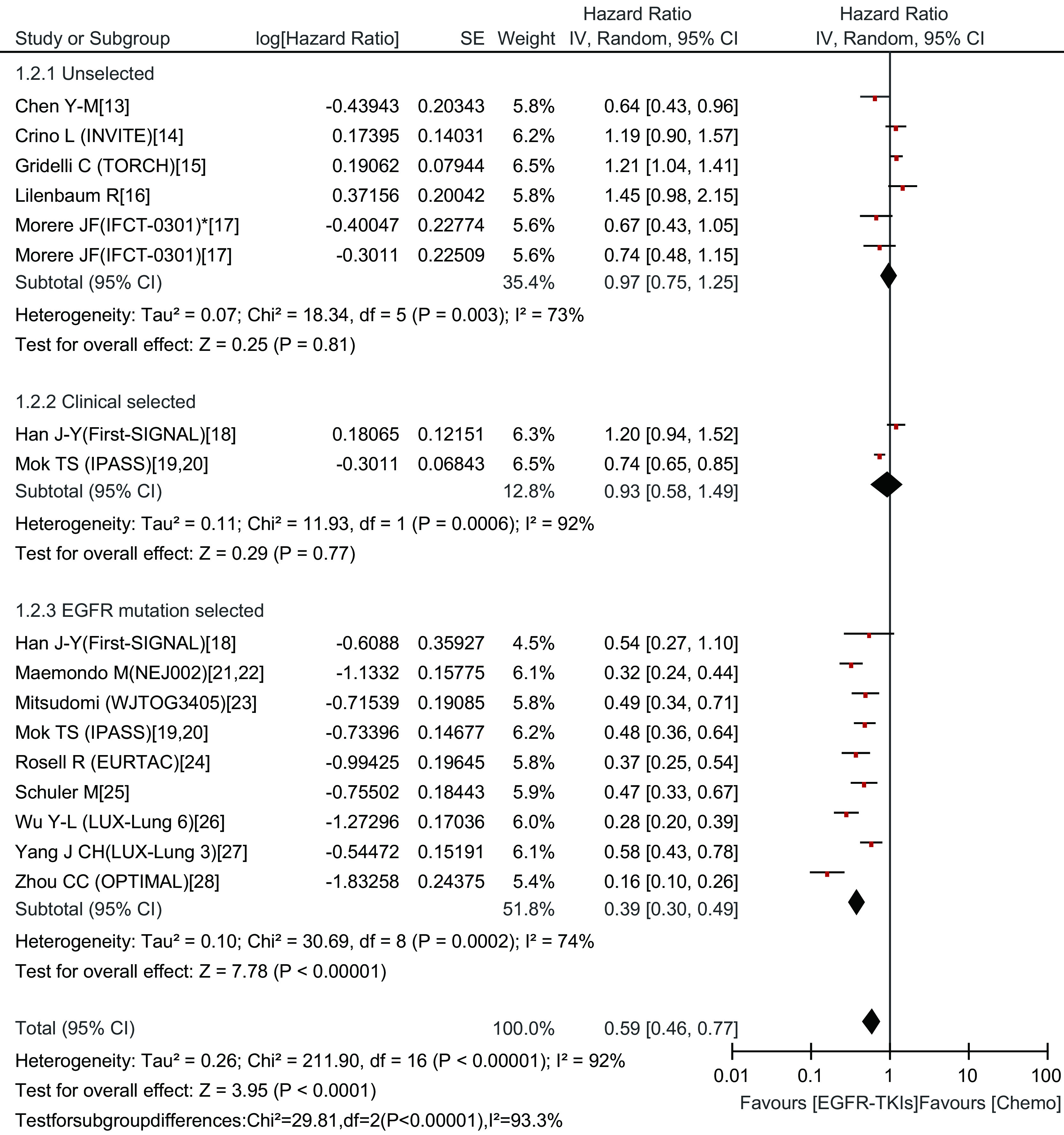
一线EGFR-TKIs治疗与化疗比较无疾病进展时间的森林图及统计学分析 Forest plot and statistics of first-line EGFR-TKI treatment versus chemotherapy for PFS. PFS: progression free survival.

#### 2.4.3 OS的*meta*分析结果

EGFR-TKIs治疗组中位OS为2.2个月-27.7个月，化疗组中位OS为3.5个月-26.6个月。*Meta*分析结果显示，总研究及各亚组同质（Chi^2^=10.97, *P*=0.28, *I*^2^=18%），采用固定效应模型。两种治疗方法在OS上并无差异（HR=0.99; 95%CI: 0.92-1.08）。亚组分析中临床选择组及*EGFR*基因突变阳性组在OS上也无差异（HR=0.91; 95%CI: 0.81-1.02）、（HR=0.99; 95%CI: 0.84-1.16），而在未选择人群，化疗比EGFR-TKIs治疗更能延长OS（HR=1.19; 95%CI: 1.02-1.40）（图 4）。

**4:**
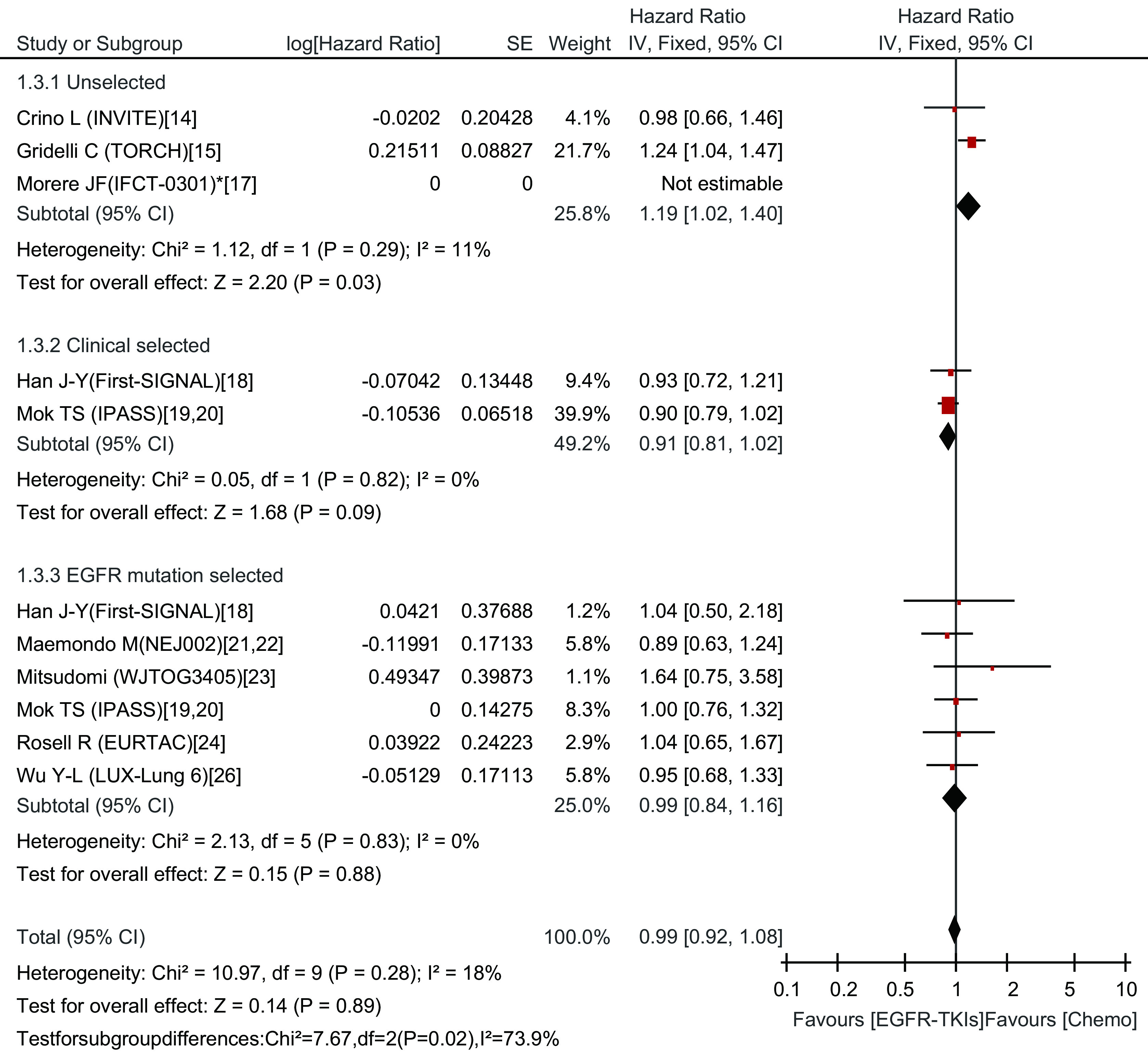
一线EGFR-TKIs治疗与化疗比较总生存期的森林图及统计学分析 Forest plot and statistics of first-line EGFR-TKI treatment versus chemotherapy for OS. OS: overall survival.

## 3 讨论

EGFR-TKIs作为一种靶向治疗药物，有较好的应用前景，但由于该药并非对所有患者均有较好疗效，所以对于不同的个体在化疗和EGFR-TKIs治疗间的选择就显得很重要，治疗的个体化或群体化，是最大限度发挥药物作用和减少医疗费用的选择。

从上述结果可知，对于*EGFR*基因突变阳性人群，EGFR-TKIs一线治疗与化疗相比有较好的有效率和延长了PFS。因此，NSCLC患者在治疗前进行基因检测是必要的，对于基因突变阳性人群建议使用EGFR-TKIs治疗。在OS方面，由于该研究为一线研究，OS容易受一线进展后治疗的影响而不能更好地反映真实情况，Broglio等^[29]^的研究指出，若一线进展后治疗时间较短，PFS延长的优势会体现在OS上，导致OS延长，且具有统计学意义，然而较长时间的一线后治疗会让研究失去OS延长的优势。此外Aboshi等^[30]^的研究认为入组患者数量（每组少于150例）、平均年龄＜63岁、男性患者比率＜70%、鳞癌所占比率＜30%会导致PFS延长的优势不能体现在OS上。

对于未选择人群，有效率及PFS在两组间都无差异，相反化疗在延长OS上更有优势。对这一人群化疗是更好的选择。在纳入的5项对未选择人群的研究中，2项研究针对老年患者，2项研究针对PS评分为2分-3分的患者，由于入选人群的基本情况较差且各研究间差异较大，会对结果产生一定影响。该亚组研究可有待更多临床研究结果出现后进一步更新。

在临床中，由于各种原因，并非所有患者都能进行基因检测，因此临床选择对于那些不能或不愿意做基因检测的人群显得至关重要。但本研究的结果并不乐观，在有效率上EGFR-TKIs治疗较化疗显示出了优势，然而在PFS和OS上，两者并无统计学上的差异。本研究中该结果还有待更新，其原因主要是首先在研究数量上，该亚组仅纳入两篇文章，有待更多该类研究出现，获取更多信息。其次，两篇文章的化疗方案并不相同，First-SIGNAL研究化疗方案为吉西他滨加顺铂，IPSS研究化疗方案为紫杉醇加卡铂。最后，从这两篇文章本身来说，存在较大的异质性。两篇文章的纳入及排除标准相似，EGFR-TKI药物相同，平均年龄相似，肿瘤分期、PS评分相同，均为亚裔人群，然而两个研究化疗周期及*EGFR*基因突变阳性率有较大差异。First-SIGNAL研究随访至患者完成9周期化疗，*EGFR*基因突变阳性率为44%^[18]^。IPSS研究随访至患者完成6周期化疗，*EGFR*基因突变阳性率为60%^[19, 20]^。从以上这些方面我们也可以看到，临床工作中对化疗方案的选择也至关重要。同时，临床选择亚组也从侧面证明了*EGFR*基因突变人群为EGFR-TKIs治疗的最适宜人群，而临床选择人群有较高的基因突变率，早在2004年Kosaka等^[31]^的研究报道了在277例患者中，111例为基因突变患者，腺癌的突变率为49%，其他病理类型只占2%；女性突变率为59%，男性为26%；不吸烟患者的突变率为66%，吸烟患者为22%。对于那些不适宜化疗而又未行基因检测的亚裔、腺癌、不吸烟患者，EGFR-TKI治疗不失为一个较好的选择。

目前，EGFR-TKI与化疗序贯治疗及联合治疗也被研究者们提出。Takeda等^[32]^的一线含铂双药化疗3周期后序贯吉非替尼治疗与含铂双药化疗6周期比较研究结果显示，序贯治疗与单纯化疗相比能延长PFS，但在OS上无明显差异，其中亚组分析显示腺癌患者序贯治疗与单纯化疗相比不仅延长了PFS还延长了OS。OuYang等^[33]^的一线化疗与EGFR-TKI联合治疗的*meta*分析结果显示，联合治疗与任一治疗相比延长了PFS，但在OS上并没有优势。相信在未来的研究中，EGFR-TKI会有更广阔的前景，发挥它们最大的优势。

综上所述，*EGFR*基因突变阳性的NSCLC患者一线EGFR-TKIs治疗比化疗拥有更好的疗效。未选择的患者若无化疗禁忌，建议行一线化疗。对于不能耐受化疗的亚裔、腺癌、不吸烟患者，一线EGFR-TKIs治疗可以短期从EGFR-TKIs的治疗中获益、远期疗效与化疗相似，因此推荐通过临床特征一线选择不能耐受化疗的NSCLC患者接受EGFR-TKIs治疗。

## References

[1] Siegel R, Ma J, Zou Z (2014). Cancer statistics, 2014. CA Cancer J Clin.

[2] D'Addario G, Früh M, Reck M (2010). ESMO Guidelines Working Group. Metastatic non-small-cell lung cancer: ESMO Clinical Practice Guidelines for diagnosis, treatment and follow-up. Ann Oncol.

[3] Soon YY, Stockler MR, Askie LM (2009). Duration of chemotherapy for advanced non-small-cell lung cancer: a systematic review and *meta*-analysis of randomized trials. J Clin Oncol.

[4] Lemmon MA, Schlessinger J (2010). Cell signaling by receptor tyrosine kinases. Cell.

[5] Arteaga CL (2001). The epidermal growth factor receptor: from mutant oncogene in nonhuman cancers to therapeutic target in human neoplasia. J Clin Oncol.

[6] Baselga J, Rischin D, Ranson M (2002). Phase Ⅰ safety, pharmacokinetic, and pharmacodynamic trial of ZD1839, a selective oral epidermal growth factor receptor tyrosine kinase inhibitor, in patients with five selected solid tumor types. J Clin Oncol.

[7] Herbst RS, Maddox AM, Rothenberg ML (2002). Selective oral epidermal growth factor receptor tyrosine kinase inhibitor ZD1839 is generally well-tolerated and has activity in non-small-cell lung cancer and other solid tumors: results of a phase Ⅰ trial. J Clin Oncol.

[8] Kris MG, Natale RB, Herbst RS (2003). Efficacy of gefitinib, an inhibitor of the epidermal growth factor receptor tyrosine kinase, in symptomatic patients with non-small cell lung cancer: a randomized trial. JAMA.

[9] Fukuoka M, Yano S, Giaccone G (2003). Multi-institutional randomized phase Ⅱ trial of gefitinib for previously treated patients with advanced non-small-cell lung cancer (The IDEAL 1 Trial) [corrected]. J Clin Oncol.

[10] Pérez-Soler R, Chachoua A, Hammond LA (2004). Determinants of tumor response and survival with erlotinib in patients with non-small-cell lung cancer. J Clin Oncol.

[11] Lynch TJ, Bell DW, Sordella R (2004). Activating mutations in the epidermal growth factor receptor underlying responsiveness of non-small-cell lung cancer to gefitinib. N Engl J Med.

[12] Ettinger DS, Akerley W, Borghaei H (2013). National comprehensive cancer network. Non-small cell lung cancer, version 2. 2013. J Natl Compr Canc Netw.

[13] Chen YM, Tsai CM, Fan WC (2012). Phase Ⅱ randomized trial of erlotinib or vinorelbine in chemonaive, advanced, non-small cell lung cancer patients aged 70 years or older. J Thorac Oncol.

[14] Crinò L, Cappuzzo F, Zatloukal P (2008). Gefitinib versus vinorelbine in chemotherapy-naive elderly patients with advanced non-small-cell lung cancer (INVITE): a randomized, phase Ⅱ study. J Clin Oncol.

[15] Gridelli C, Ciardiello F, Gallo C (2012). First-line erlotinib followed by second-line cisplatin-gemcitabine chemotherapy in advanced non-small-cell lung cancer: the TORCH randomized trial. J Clin Oncol.

[16] Lilenbaum R, Axelrod R, Thomas S (2008). Randomized phase Ⅱ trial of erlotinib or standard chemotherapy in patients with advanced non-small-cell lung cancer and a performance status of 2. J Clin Oncol.

[17] Morère JF, Bréchot JM, Westeel V (2010). Randomized phase Ⅱ trial of gefitinib or gemcitabine or docetaxel chemotherapy in patients with advanced non-small-cell lung cancer and a performance status of 2 or 3 (IFCT-0301 study). Lung Cancer.

[18] Han JY, Park K, Kim SW (2012). First-SIGNAL: first-line single-agent iressa versus gemcitabine and cisplatin trial in never-smokers with adenocarcinoma of the lung. J Clin Oncol.

[19] Mok TS, Wu YL, Thongprasert S (2009). Gefitinib or carboplatin-paclitaxel in pulmonary adenocarcinoma. N Engl J Med.

[20] Fukuoka M, Wu YL, Thongprasert S (2011). Biomarker analyses and final overall survival results from a phase Ⅲ, randomized, open-label, first-line study of gefitinib versus carboplatin/paclitaxel in clinically selected patients with advanced non-small-cell lung cancer in Asia (IPASS). J Clin Oncol.

[21] Maemondo M, Inoue A, Kobayashi K (2010). Gefitinib or chemotherapy for non-small-cell lung cancer with mutated EGFR. N Engl J Med.

[22] Inoue A, Kobayashi K, Maemondo M (2013). Updated overall survival results from a randomized phase Ⅲ trial comparinggefitinib with carboplatin-paclitaxel for chemo-naive non-small cell lung cancer with sensitive EGFR gene mutations (NEJ002). Ann Oncol.

[23] Mitsudomi T, Morita S, Yatabe Y (2010). Gefitinib versus cisplatin plus docetaxel in patients with non-small-cell lung cancer harbouring mutations of the epidermal growth factor receptor (WJTOG3405): an open label, randomised phase 3 trial. Lancet Oncol.

[24] Rosell R, Carcereny E, Gervais R (2012). Erlotinib versus standard chemotherapy as first-line treatment for European patients with advanced EGFR mutation-positive non-small-cell lung cancer (EURTAC): a multicentre, open-label, randomised phase 3 trial. Lancet Oncol.

[25] Schuler M, Yang JCH, Sequist LV (2013). Efficacy of afatinib vs. chemotherapy in treatment-na?ve patients with nonsmall cell lung cancer (NSCLC) harbouring activating *EGFR* mutations with or without metastatic brain disease. J Thorac Oncol.

[26] Wu YL, Zhou C, Hu CP (2014). Afatinib versus cisplatin plus gemcitabine for first-line treatment of Asian patients with advanced non-small-cell lung cancer harbouring *EGFR* mutations (LUX-Lung 6): an open-label, randomised phase 3 trial. Lancet Oncol.

[27] Yang J C-H, Schuler MH, Yamamoto N (2012). LUX-Lung 3: A randomized, open label, phase Ⅲ study of afatinib versus pemetrexed and cisplatin as first-line treatment for patients with advanced adenocarcinoma of the lung harboring *EGFR*-activating mutations. J Clin Oncol.

[28] Zhou CC, Wu YL, Chen G (2011). Erlotinib versus chemotherapy as first-line treatment for patients with advanced *EGFR* mutation-positive non-small-cell lung cancer (OPTIMAL, CTONG-0802): a multicentre, open-label, randomised, phase 3 study. Lancet Oncol.

[29] Broglio KR, Berry DA (2009). Detecting an overall survival benefit that is derived from progression-free survival. J Natl Cancer Inst.

[30] Aboshi M, Kaneko M, Narukawa M (2014). Factors affecting the association between overall survival and progression-free survival in clinical trials of first-line treatment for patients with advanced non-small cell lung cancer. J Cancer Res Clin Oncol.

[31] Kosaka T, Yatabe Y, Endoh H (2004). Mutations of the epidermal growth factor receptor gene in lung cancer: biological and clinical implications. Cancer Res.

[32] Takeda K, Hida T, Sato T (2010). Randomized phase Ⅲ trial of platinum-doublet chemotherapy followed by gefitinib compared with continued platinum-doublet chemotherapy in Japanese patients with advanced non-small-cell lung cancer: results of a west Japan thoracic oncology group trial (WJTOG0203). J Clin Oncol.

[33] OuYang PY, Su Z, Mao YP (2013). Combination of EGFR-TKIs and chemotherapy as first-line therapy for advanced NSCLC: a *meta*-analysis. PLoS One.

